# An Optimal Protocol to Analyze the Rat Spinal Cord Proteome

**DOI:** 10.4137/bmi.s2965

**Published:** 2009-10-28

**Authors:** F. Gil-Dones, S. Alonso-Orgaz, G. Avila, T. Martin-Rojas, V. Moral-Darde, G. Barroso, F. Vivanco, J. Scott-Taylor, M.G. Barderas

**Affiliations:** 1 Department of Vascular Pathophysiology, Hospital Nacional de Paraplejicos (HNP), SESCAM, Toledo; 2 Sensorimotor Function Group, Hospital Nacional de Paraplejicos (HNP), SESCAM, Toledo; 3 Proteomics Unit, Hospital Nacional de Paraplejicos (HNP), SESCAM, Toledo; 4 Department of Immunology, Fundacion Jimenez Diaz, Madrid; 5 Department of Biochemistry and Molecular Biology I, Universidad Complutense, Madrid. Email: megonzalezb@sescam.jccm.es

**Keywords:** proteomics, two dimensional electrophoresis (2-DE), Liquid Chromatography Mass Spectrometry/Mass Spectrometry (LC-MS MS), spinal cord, proteome

## Abstract

Since the function of the spinal cord depends on the proteins found there, better defing the normal Spinal Cord Proteome is an important and challenging task. Although brain and cerebrospinal fluid samples from patients with different central nervous system (CNS) disorders have been studied, a thorough examination of specific spinal cord proteins and the changes induced by injury or associated to conditions such as neurodegeneration, spasticity and neuropathies has yet to be performed. In the present study, we aimed to describe total protein content in the spinal cord of healthy rats, employing different proteomics tools. Accordingly, we have developed a fast, easy, and reproducible sequential protocol for protein extraction from rat spinal cords. We employed conventional two dimensional electrophoresis (2DE) in different pH ranges (eg. 4–7, 3–11 NL) combined with identification by mass spectrometry (MALDI-TOF/TOF), as well as first dimension protein separation combined with Liquid Chromatography Mass Spectrometry/Mass Spectrometry (LC-MS/MS) to maximise the benefits of this technology. The value of these techniques is demonstrated here by the identification of several proteins known to be associated with neuroglial structures, neurotransmission, cell survival and nerve growth in the central nervous system. Furthermore this study identified many spinal proteins that have not previously been described in the literature and which may play an important role as either sensitive biomarkers of dysfunction or of recovery after Spinal Cord Injury.

## Introduction

Spinal cord injury (SCI) has a significant disabling and lifelong effect on many people and as such, it represents a major challenge for successful health care management. SCI is a devastating neurotrauma insult that can lead to the loss of sensory and motor function below the level of injury.^1^, ^2^ The progressive pathological changes initiated by SCI include complex and evolving molecular cascades whose interrelationships are not fully understood, and many molecules involved in these processes remain to be discovered.^3^–^7^ To date, brain and cerebrospinal fluid samples from patients with different central nervous system (CNS) disorders have been studied extensively using different biochemical assays.^8^–^12^ However, relatively few studies have focused on spinal cord protein content, and the changes induced after spinal neurotrama or in association with symptoms such as spasticity or neuropathic pain. Indeed, recent studies have been conducted to screen for a wide range of proteins following SCI using comparative proteomic technologies.^13^–^17^

The tremendous advances in molecular biology, mainly in the field of genomics and proteomics, open the possibility to understand the mechanisms underlying many neuropathologies. After genomics, proteomics is often considered the next logical step to study biological systems, with the added capacity to describe the spatiotemporal differences in protein expression, both in normal and pathological tissue.^18^–^20^ The proteome represents all the proteins expressed by a genome, cell, tissue or organism at a given time under defined physiological conditions. Since most physiological body functions reflect the integrity of their proteins, understanding the complex biological processes active in the spinal cord during pathological conditions like SCI requires the key proteins involved at an early stage of the neurotrauma^21^, ^22^ (acute phase) and during injury progression to be identified.

Proteomic analysis is now a key biomedical tool to establish protein maps that can assist in biomarker discovery and in the identification of therapeutic targets. In this respect, an important and challenging task is to develop protocols designed to extend our knowledge of the spinal cord (SC) protein profile that combine mass spectrometry with two dimensional gels (2-DE). Until now most studies have focussed on one protein or on a small number of proteins using standard techniques such as Western blotting, immunohistochemistry or RT-PCR, which fail to provide complete information regarding the general physiological state of the SC. In contrast, proteomic analysis is useful as multiple molecules can be assayed simultaneously using separation techniques combined with the powerful new mass spectrometry technologies, such as MALDI-TOF/TOF (Matrix Assisted Laser Desorption Ionization-Time of Flight/Time of Flight Mass Spectrometry), SELDI-TOF (Surface Enhanced Laser Desorption Ionization Time Of Flight Mass Spectrometry), Protein Arrays, LCM (Laser Capture Microdissection), MS-Imaging, LC-MS (Liquid Chromatography Mass Spectrometry), TOF-SIMS (Time of Flight Secondary Ion Mass Spectrometry).^23^–^29^

However, the development of global protein analysis using proteomic technologies needs to address several limitations and challenges. An important tool applied to study the proteome is 2-DE, whereby proteins are first separated by isoelectric focusing (IEF) and then based on their molecular weight by SDS-PAGE (sodium dodecyl sulphate polyacrylamide gel electrophoresis).^30^–^32^ However, this technique presents some important limitations that could be resolved by the application of other proteomics tools such as LC-MS/MS.^33^ In addition, there is a need to develop efficient protocols to extract most of the proteins present in the spinal cord, given the limitations of each technique and the complexity of the proteome.

**Figure 1.**
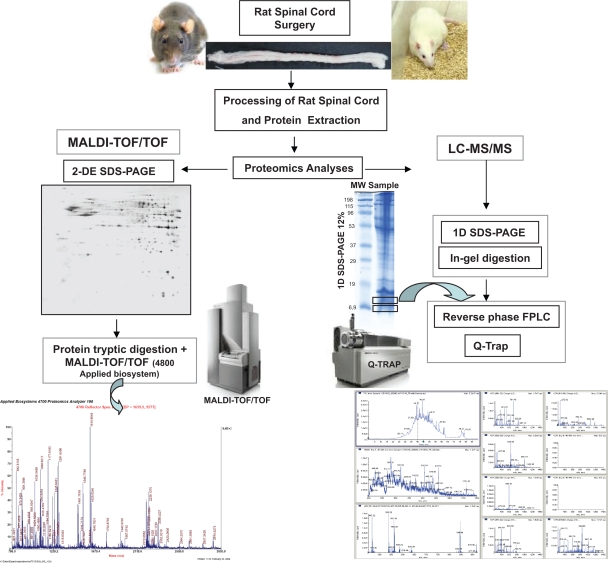
The proteomic platforms used in this study and a flowchart demonstrating the strategy for the rat spinal cord analysis. Schematic illustration of the proteomics methods used to characterise the rat spinal cord proteome.

In this technical report, we present a fast, easy and reproducible protocol to extract SC proteins and analyze its proteome (Fig. 1). The aim of this study is to describe the majority of the proteins extracted from the rat SC proteome by employing conventional 2-DE spot maps over different pH ranges and MALDI-TOF/TOF for their identification, in combination with LC-MS/MS to maximise the utility of this technology. The application of this newly developed optimal protein extraction protocol compatible with 2-DE and LC-MS/MS will permit future translational studies to identify the main pathophysiological mechanisms associated with SCI.

## Materials and Methods

### Collection of rat spinal cords

Thoracico-lumbar spinal cord tissue was obtained from 12 week old male adult Wistar rats (n = 6: Harlan SA, Milano, Italy) weighing between 300–400 g sacrificed with an intraperitoneal overdose of Sodium Pentobarbital (Dolethal, Norman SA). Shortly afterwards, the spinal cord tissue was extracted using hydraulic pressure applied to the caudal vertebral canal, whereupon the tissue was cleaned with a saline solution (0.9%). The thoracico-lumbar segments were carefully dissected out and then frozen and stored at −20 °C until analyses.

### Rat spinal cord processing: protein extraction

**Figure 2.**
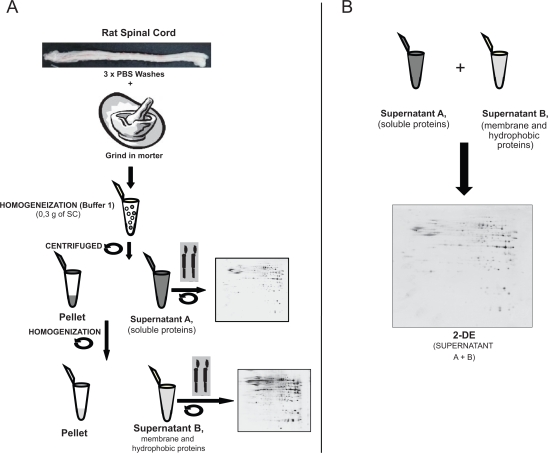
The protocol to extract proteins from the rat spinal cord. **A**) After surgery the spinal cord tissue was washed in saline buffer to eliminate blood contaminants and tissue was homogenized **(Buffer 1)** and later a new extraction of proteins was realized using buffer 2. Supernatant A, containing most of the soluble proteins and supernatant B, containing membrane and hydrophobic proteins were analysed separately in 2-DE in order to check the efficiency of the protein extraction protocol. **B**) Supernatant A and B were mixed and analysed by 2-DE.

After removal from −20 °C storage, the tissue was maintained at 4 °C in PBS solution and all the following steps in the protocol were performed at 4 °C (Fig. 2).

Firstly, the tissue was washed 3 times in PBS to remove blood contaminants and it was then ground into a powder with a mortar in Liquid Nitrogen. This powder (0.3 g) was resuspended in 300 μL of protein extraction buffer 1 (Tris 10 mM [pH 7.5], 500 mM NaCl 0.1%, Triton x-100, 1% β-mercaptoethanol and 1 mM PMSF).^34^ The homogenate was sonicated for 5 minutes and centrifuged at 21,000 g (5840 R Eppendorf) for 15 minutes at 4 °C to precipitate the membrane and tissue debris. The supernatant (supernatant A), containing most of the soluble proteins was collected and stored at 4 °C. The pellet was then dissolved in a buffer containing 7 M Urea, 2 M Thiourea, 5% CHAPS,^35^, ^36^ and it was again centrifuged at 21,000g to obtain a second supernatant separated from the pellet of tissue debris (Supernatant B), mainly composed of membrane proteins. The tissue debris was then resuspended in protein loading buffer (Tris 0.5 M [pH 8.0], SDS 10%, Glycerol, β-mercaptoethanol and bromophenol blue 0.02%) and the protein concentration was determined by the Bradford-Lowry method using the Bio-Rad protein assay commercial Kit.^37^ Finally, the protein composition was analyzed by resolving 25 μg of total protein content from each sample by SDS-PAGE 12% (Acrylamide/Bisacrylamide 30%/0.8% v/v).

### Two-dimensional electrophoresis (2-DE)

All chemicals and instruments used for 2-DE gels have been described previously.^35^, ^36^ Both the soluble and hydrophobic protein extracts were mixed and dialysed against 2 mM Tris buffer using Mini dialysis Kit 1 kDa cut-off (GE Healthcare). Subsequently, 300 μg of each protein extract was cleaned with the 2 D Clean up Kit (GE-Healthcare) and resuspended in rehydration buffer (7 M Urea, 2 M Thiourea, 4% CHAPS, 1%–2% Ampholites and 1% TBP: Bio-Rad). Isoelectric focusing (IEF) was performed in an IPGphor unit (GE Healthcare). The strips (17 cm and pH 4–7: Bio-Rad, or 24 cm pH 3–11 NL—non-lineal: GE Healthcare) were actively rehydrated at 20 °C for 12 h at 50 V to enhance protein uptake, and the voltage was then increased according to the following program: 500 V for 30 minutes, 1000 V for 1 h, 1000–2000 V in 1 h (gradient), 2000–5000 V in 2 h (gradient), 5000–8000 V in 1 h (gradient), 8000 V to a total 88,000 V/h.

Subsequently, the strips IEF were equilibrated as described previously^35^, ^36^ and the second dimension (SDS-PAGE) was run according to Laemmli’s method,^38^ using a Protean II system (Bio-Rad) at 1 W/gel at 20 °C overnight. Gels were fixed and stained by Silver Staining (GE Healthcare, according to the manufacturer’s instructions) and they were then scanned with a GS-800 Calibrated Densitometer (Bio-Rad). Evaluation of the 2-DE gels was performed using PDQuest 2DE Gel Analysis Software version 8.0.1 (Bio-Rad). Reproducibility was tested comparing the variation within the different gels in the same group using the same software.

### In gel digestion

Spots (200) were manually excised, automatically digested with “Ettan Digester” (GE Healthcare) and identified at the HNP Proteomic Unit according to Schevchenko et al^39^ with minor modifications.^40^ Gel plugs were reduced with 10 mM dithiothreitol (Sigma Aldrich) in 50 mM ammonium bicarbonate (99% purity; Scharlau) and by alkylation with 55 mM iodoacetamide (Sigma Aldrich) in 50 mM ammonium bicarbonate. The gel fragments were then rinsed with 50 mM ammonium bicarbonate in 50%. Methanol (gradient, HPLC grade, Scharlau) and acetonitrile (gradient, HPLC grade, Scharlau), and they were dried in a Speedvac. Modified porcine trypsin (sequencing grade; Promega, Madison, WI, USA) was added to the dry gel pieces at a final concentration of 20 ng/μl in 20 mM ammonium bicarbonate and the digestion proceeded at 37 °C overnight. Finally, 70% aqueous acetonitrile and 0.1% formic acid (99.5% purity; Sigma Aldrich) was added for peptide extraction.

### Protein identification by MALDI-TOF/TOF

An aliquot of each digestion was mixed with an aliquot of the matrix solution (3 mg/mL α-cyano-4-Hydroxycinnamic acid: Sigma Aldrich) in 30% ACN, 15% 2-propanol and 0.1% TFA. This mixture was pipetted directly onto the stainless steel sample plate of the mass spectrometer (384 Opti-TOF 123 × 81 mm MALDI: Applied Biosystem) and dried at room temperature.

The MALDI-MS/MS data were obtained in an automated analysis loop using a 4800 Plus MALDI TOF/TOF Analyzer (Applied Biosystems). Spectra were acquired in the reflector positive-ion mode with a Nd:YAG laser (355 nm wavelength at a frequency of 200 Hz), and between 100 and 2000 individual spectra were averaged. The experiments were acquired in a uniform mode with a fixed laser intensity. For the MS/MS 1 kV analysis mode, precursors were accelerated to 8 kV in source 1, and they were selected at a relative resolution of 350 (FWHM) with metastable suppression. Fragment ions generated by collision with air in a CID chamber were further accelerated at 15 kV in source 2. Mass data was analysed automatically with the 4000 Series Explorer Software version 3.5.3 (Applied Biosystems). Internal calibration of MALDI-TOF mass spectra was performed using two trypsin autolysis ions with m/z = 842.510 and m/z = 2211.105. For calibration in the MS/MS mode, the fragment ion spectra obtained from Glub-fibrinopeptide were used (4700 Cal Mix, Applied Biosystems). MALDI-MS and MS/MS data were combined through the GPS Explorer Software Version 3.6 to search a nonredundant protein database (Swissprot 56.7) using the Mascot software (version 2.2, Matrix Science), employing the following parameters: 50 ppm precursor tolerance; 0.6 Da MS/MS fragment tolerance; and allowing 1 missed cleavage, carbamidomethyl cysteines and methionine oxidation as a modification. The MALDI-MS/MS spectra and database search results were manually inspected in detail using the aforementioned software.

### LC-MS/MS and database searching

#### Sample preparation

Total spinal cord proteins (50 μg) were resolve by one dimensional (1-D) SDS-PAGE 12%. Each lane in the 1-D gel was divided into 24 gel slices that were manually excised and then digested automatically using the Ettan Digester (GE Healthcare). The digestion was performed according to Schevchenko et al^39^ with minor modifications^40^ and using Modified porcine trypsin (sequencing grade; Promega, Madison, WI, USA) diluted to a final concentration of 20 ng/μl in 20 mM ammonium bicarbonate. The gel slices were incubated with 10 mM dithiothreitol (Sigma Aldrich) in 50 mM ammonium bicarbonate (99% purity; Scharlau) for 30 minutes at 56 °C and after reduction, they were alkylated with 55 mM iodoacetamide (Sigma Aldrich) in 50 mM ammonium bicarbonate for 20 minutes at RT. Gel plugs were washed with 50 mM ammonium bicarbonate in 50% methanol (gradient, HPLC grade, Scharlau), rinsed in acetonitrile (gradient, HPLC grade, Scharlau) and dried in a Speedvac. Dry gel pieces were then embedded in sequencing grade modified porcine trypsin (20 ng/μL: Promega, Madison, WI, USA) and after digestion at 37 °C overnight, the peptides were extracted with 70% acetonitrile (ACN) in 0.1% formic acid (99.5% purity; Sigma Aldrich). Finally, the samples were dried in a speedvac and resuspended in 98% water with 0.1% formic acid (FA) and 2% ACN.

#### LC-MS/MS and database searching

The LC/MSMS system was comprised of a TEMPO nano LC system (Applied Biosystems) combined with a nano LC Autosampler. Each sample was injected in three replicates (3 μL) using mobile phase A (2% ACN/98% water, 0.1% FA) at a flow rate of 10 μL/minute for 10 minutes. Peptides were loaded onto a μ-Precolumn Cartridge (Acclaim Pep Map 100 C18, 5 μm, 100Å; 300 μm i.d. × 5 mm, LC Packings) to preconcentrate and desalt samples. The RPLC was performed on a C18 column (Acclaim Pep Map 100 C18, 3 μm, 100Å; NAN75-15-03-C18PM, 75 μm I.D. × 15 cm, LC Packings) using mobile phase A (2% ACN/98% water, 0.1% FA) and mobile phase B (98% ACN/2% water, 0.1% FA). Peptides were eluted at a flow rate of 300 nL/minute over the following gradient: initial conditions of 5% B that increased to 50% B over 70 minutes, 50 to 95% B for 1 minute and then 95% B for 3 minutes, returning to the initial conditions (5% B) over 2 minutes and maintaining these conditions for a further 14 minutes.

The LC-MS/MS analysis was performed on an AB/MDS Sciex 4000 Q TRAP System with NanoSprayII Source (Applied Biosystems). The TEMPO nano LC system and 4000 QTRAP were both controlled by Analyst Software v.1.4.2.

All the MS and MS/MS data were obtained in positive ion mode, with an ion spray voltage of 2800 V and a declustering of 85V. Nanoflow interface was heated at 150 °C, and the source gas 1 and curtain gas were set to 20 and 10, respectively. Nitrogen was applied as both curtain and collision gas. An Information Dependent Acquisition (IDA) method was programmed, with a full scan Enhanced MS (EMS) experiment at 4000 amu/s for ion profiling that was followed by an enhanced resolution (ER) MS experiment at 250 amu/s. The ER experiment permitted charge state recognition that was further submitted to IDA criteria to select precursor ions, and to estimate the collision energy to fragment them. These IDA criteria were set to select the 8 most intense double, triple or quadruple charged ions from 400–1200 m/z that exceed 100,000 counts for fragmentation in the LINAC collision cell. Isotopes within a 4.0 amu window and with a mass tolerance of 1,000,000 mmu were excluded. These 8 ions were submitted to 8 independent Enhanced Product Ion (EPI) MS/MS experiments at 4000 amu/s with Dynamic Fill Time (DFT). The total number of MS and MS/MS experiments per cycle was 10 (1 EMS, 1 ER and 8 EPI), resulting in a total cycle time of 5.0058 s.

Analyst software creates wiff format files including all the spectra data that were batch-processed with ProteinPilot^™^ Software 2.0.1 (Applied Biosystems/MDS Sciex). This software automatically generated peak lists that were searched against the Swissprot database version 56.7 using Paragon Algorithm (Applied Biosystems). Settings in the Paragon Algorithm included a detected protein threshold >1.0 (90%), Iodoacetamide was selected for Cys alkylation and Gel-based ID was selected as a special factor.

## Results

### 

#### 

##### Rat spinal cord processing and protein extraction

To describe the complete proteome of an organ or tissue, it is necessary to establish an efficient extraction protocol to maximize protein recovery. Here, we present a flowchart to explain our approach to the proteomic study of rat SC (Fig. 1) and a schedule of the consecutive extraction protocol (Fig. 2). This method was based on two consecutive steps using two distinct extraction buffers, the first of which extracted the more soluble proteins, while the second was designed to dissolve the membrane and hydrophobic proteins that were assumed to be abundant in SC tissue.

##### Sample preparation and conventional 2-DE

**Figure 3.**
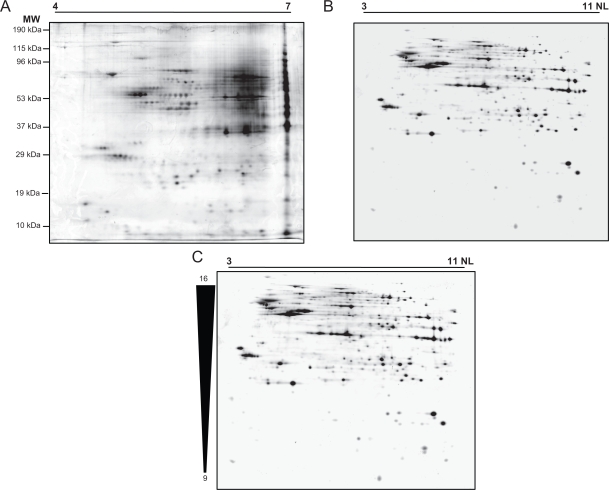
2-DE gel images. 2-DE was performed with IPG strips at different pH ranges: **A**) pH 4–7 (left) and **B**) pH 3–11 NL (right). **C**) 2-DE gel performed with 3–11 NL IPG strip and 9%–16% acrylamide/bisacrylamide.

In order to reduce the presence of lipids and other interfering substances, samples were sonicated, filtered with a micro spin-filter (SIGMA) and cleaned with the Clean-up Kit (GE Healthcare). We tested different pH ranges (pH 4–7; pH 3–11 NL) in order to select that which was optimal to detect the maximal number of spots with the greatest resolution. Spinal Cord protein extracts were quantified and approximately 300 μg was loaded onto each 2-DE gel. After analysis with the PD-Quest software (Bio-Rad), around 300 spots were detected by 2-DE in the 4–7 pH range (Fig. 3A). However, these gels did not present an homogeneous spot distribution due to the fact that most of them co-localized in the same area.

For this reason, we performed 2-DE gels with 24 cm pH 3–11 NL IPG strips. We obtained a good distribution, definition and a large number of spots under these conditions, although some streaking in the 53–96 kDa molecular weight region could be due to the high concentration of these abundant proteins. This problem did not arise in the same region of the pH 4–7 2 D gels. Hence, the use of the two types of gels with complementary pH ranges (pH 4–7 and 3–11 NL) helped improve the overall spot resolution, as reported previously.^35^

Thus, more than 1000 spots were detected after PD-Quest software analysis, improving the resolution and permitting the subsequent identification of the spots (Fig. 3B). Reproducibility was tested by comparing the variation within the different gels in the same group using the PD Quest 8.0 software. An analysis of 1126 spots revealed a coefficient of variation (CV) < 50% for 90.4% of the spots in same group of gels. Among these, a CV < 30% was obtained for 67.1% of the spots. These data confirmed the high reproducibility of the gels obtained with the method used.

##### Protein identification (MALDI-TOF/TOF)

**Figure 4.**
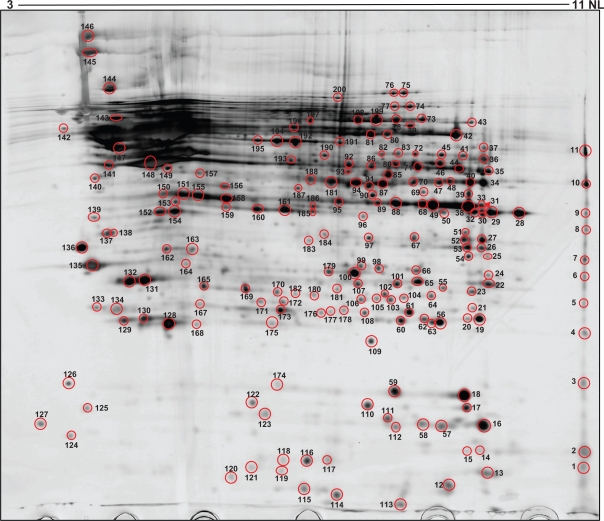
Preparative 2-DE Gel (700 μg). Spot Map of the proteins identified. The characterization of the spots identified is shown in Table 1.

In order to verify the effectiveness of our methodology, 200 spots were chosen at random, they were excised from the stained 2-DE gels, digested and the resultant tryptic peptides were deposited an a MALDI plaque and applied in a 4800 Plus MALDI-TOF/TOF Analyzer (Applied Biosystem). Proteins were identified by Peptide mass fingerprinting using the “MASCOT” search engine (www.matrixscience.com). All the spots were identified and they corresponded to 128 proteins (Fig. 4), as summarized in Table 1 where their molecular weight, isoelectric point, cellular sublocalization and function are shown.

Our data show the broad range of proteins identified by 2-DE from Macrophage migration inhibitory factor 12.5 kDa up to the Neurofilament heavy polypeptide with a molecular weight of 115.31 kDa. Furthermore we identified the Myelin basic protein, as the most basic protein (pI 11.25) and Calreticulin as the most acidic (pI 4.33).

##### Liquid-Chromatography Mass Spectrometry (LC-MS/MS)

To improve the number of proteins identified by MALDI, a LC-MS analysis was carried out. Total rat SC protein (50 μg) was resolved by SDS-PAGE and after Coomassie staining (PageBlue^™^ Protein Staining Solution, Fermentas), the gel was divided and cut into 24 pieces, each of which was subjected to in-gel tryptic digestion. After digestion, the peptide samples were analyzed by HPLC (TEMPO, Applied Biosystem) and the peptides eluted were analyzed on a Q-TRAP ion trap MS workstation (Applied Biosystem).

These analyses identified a total of 18,734 peptides that corresponded to 41,481 spectra. After data grouping and filtration, 387 proteins were identified (cut off > 1 and 90% of confident) and their theoretical MW, pI, subcellular localization and function are shown in Table 2, excluding the proteins previously identified by 2-DE. Many acidic proteins were identified, such as Acidic leucine-rich-nuclear phosphoprotein 32 family member B with a pI of 3.87, and basic proteins such as Myelin basic protein with a pI of 11.25. The molecular weights of these proteins ranged from 299.53 kDa for the Microtubule-associated protein 1A to 7850.14 Da for the gamma-2 subunit of the Guanine nucleotide-binding protein G(I)/G(S)/G(O).

##### Characterization and classification of the proteins identified

**Figure 5.**
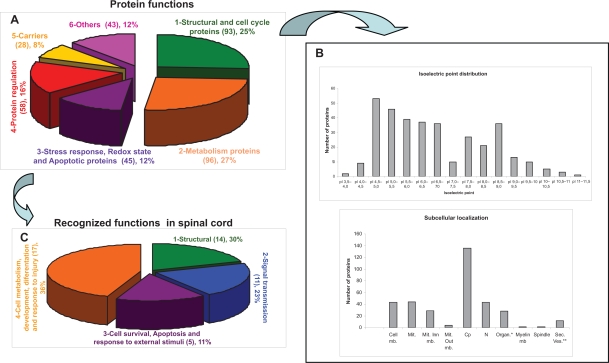
Characterization of the spinal cord proteins identified. **A**) The functional grouping of all the proteins identified using 2-DE and MALDI-TOF/TOF together with LC-MS/MS are presented. **B**) Isoelectric point distribution and subcellular localization of the proteins identified. **C**) Additional classification of the proteins with recognized function in spinal cord.

The proteins identified by MALDI-TOF/TOF and LC-MS/MS were characterized according to their molecular weight (MW), isoelectric point (pI), subcellular localization and recognized function. In total 367 unique proteins were identified with the different techniques employed. On the basis of Swiss-Prot and NCBI database information, the proteins were classified into six functional groups (Fig. 5A): Structural and Cell Cycle Proteins; Metabolic Proteins; Stress Response, Redox State and Apoptosis Proteins; Regulation proteins; Carriers and Other proteins. The different types of protein functions assigned to the proteins identified and the relative proportion of each group were represented (Fig. 5A represents), and a graph of the distribution of pI’s and cellular localization was generated (Fig. 5B). In addition, similar graphs were generated to represent the same features of those proteins recognized to be active in the nervous system.

## Discussion

To understand the complex biological processes at play in the central nervous system the key proteins involved must be identified. The exploration of the proteome has attracted increasing interest in recent years, particularly to establish reference maps designed to assist in biomarker discovery. In this regard, defining the complete spinal cord proteome is still an important challenge. This proteome may represent a fundamental key to better understand normal spinal cord physiology, as well as providing important clues to discover the molecular basis of neurodegeneration after spinal cord injury.

In the present study, we have described the proteins present in the rat spinal cord by employing different proteomic tools. Accordingly, we have defined a fast, easy and reproducible protein extraction protocol for the spinal cord. Efficient protein extraction is an essential step in proteomic studies, and the development of this specific sequential extraction augmented the number of proteins isolated, focusing mainly on membrane and hydrophobic proteins. As expected, we identified many mitochondrial and membrane proteins, as well as many soluble proteins, further supporting the efficiency of this methodology.

One of the major problems associated with proteomic analyses are the contaminants in the sample that could interfere with the isoelectrofocusing of spinal proteins (salts, DNA, lipids …). To diminish the effect of this interference, a filter step was included before initiating the 2-DE gel protocol. We employed conventional 2-DE over different pH ranges (e.g. 4–7 and 3–11 NL) to generate different maps that could help search for potential biomarkers. Furthermore, the high degree of 2-DE gel reproducibility and the resolution obtained is necessary to generate good quality maps from the rat spinal cord and for future differential expression analyses. The gels focused with 17 cm pH 4–7 IPG strips did not resolve a large number of spots, and some proteins with a high isoelectric point were not focused correctly with a line of precipitated proteins appearing at the basic extreme of the gel. This distribution in 2-DE gels pH 4–7 could present problems for posterior spot identification, and even for future differential expression analyses between healthy individuals and patients. Accordingly, better resolution was obtained with 2-DE gels with non-linear pH3–11 24 cm IPG strips, avoiding the precipitation of basic proteins. These quality of these gels was relatively high and with a good protein spot distribution, leading to the identification of 200 different spots by MALDI-TOF/TOF.

It is important to note that 2-DE gels cannot resolve proteins below 10 kDa and above 100 kDa, including the more acidic or basic proteins. To maximize the number of proteins identified and to complement the results obtained for 2-DE MALDI-MS/MS, LC-MS/MS analyses identified a further 367 unique proteins. Interestingly both proteomic tools could detect proteins with a broad range of molecular weights and isoelectric points, reflecting the efficiency of the methods employed. We found many proteins in the rat spinal cord with theoretical isoelectric points between 4.0–6.0 and 8.0–9.5, although less were obtained between 6.5 and 7.5.

The spinal proteins were classified into 6 different functional groups: Structural and Cell Cycle Proteins (25%), Metabolic Proteins (30%), Stress Response, Redox State and Apoptosis Proteins (16%), Regulation proteins (8%), Carriers and Other proteins Structural Proteins 12%. Structural and cell cycle proteins constituted a complex and heterogeneous group of cytoskeleton proteins, such as Microtubule-associated protein 1A, myelin sheet, or extracellular matrix and attachment proteins. In addition, DNA scaffold proteins and other structural proteins implicated in mitotic division and cell cycle regulation were characterized, making up around 25% of the total proteins identified. The second category, metabolic proteins, was also very broad and it reached nearly 30% of the total protein content, mainly containing hydrolytic and glucolytic enzymes. The third group, Stress Response, Redox State and Apoptosis proteins, was also a complex group made up of different proteins implicated in stress and injury response (Heat Shock Proteins). Furthermore, we included other proteins here associated with reducing oxidative damage and apoptosis. This group contained around 12% of the total proteins identified. Regulatory proteins related to protein synthesis, including transcription and translation, protein folding and degradation, made up about 16% of the proteins identified. Protein carriers were comprised of transporters and other metabolite binding molecules that represented approximately 8% of the total. Finally, a category of proteins that could not be classified into any of the above groups was denominated as “other” and contributed up to 12% to the complete proteome described here.

The proteins identified with a recognized function in the SC were organized into four functional groups. The numerous proteins in each functional group suggests that the technique developed in this report will be extremely useful to identify possible therapeutic targets for spinal cord injury, and pathways that may arrest the development of associated pathologies such as neuropathic pain and spasticity. Furthermore this technique will be important to develop future regenerative strategies.

Structural proteins were defined that included many common neuronal and glial proteins normally present in central nervous system tissue such as: Neurofilament (NF), Glial fibrillary acidic protein (GFAP), Myelin basic protein (MBP), Myelin-associated glycoprotein (MAG), Neural cell adhesion molecule (NCAM) and Macrophage migration inhibitory factor (MIF). Several of these proteins have a clear role during acute SCI such as GFAP in gliogenesis^41^ or MIF in astrocyte proliferation,^42^ while an increase in MAG would suggest the presence of a spinal environment that is inhibitory to nerve growth.^43^

The second group of proteins were related to neurotransmission. Several Vesicle-associated membrane proteins (VAMPs) were identified but only some of these are thought to be upregulated in the pathological state following SCI, although similar changes may have been identified following peripheral nerve injury axotomy.^44^ Many others were related to glutamatergic communication such as Glutamine synthetase (GS) and Glutamate dehydrogenase (GDH). These two proteins are known to be therapeutic targets for the successful treatment of spinal cord ischemia.^45^

Among the proteins responsible for cell survival and combating apoptosis, the presence of Gamma-enolase, Glucose-6-phosphate isomerase, Peroxiredoxin-2 (possible anti-oxidant protein) and Protein DJ-1 in the normal spinal cord should be highlighted, as opposed to only one protein (Glyceraldehyde-3-phosphate dehydrogenase) associated with a pro-apoptotic profile. The upregulation of neuron-specific enolase has been previously described as a potential biomarker of acute SCI.^46^ An increase in glyceraldehyde-3-phosphate dehydrogenase in spinal cord tissue has been demonstrated after contusion injury,^47^ while previous proteomic analysis has highlighted the upregulation of peroxiredoxin 2 protein after experimental SCI.^48^

Lastly, numerous proteins associated with cell metabolism, development, and response to injury were identified, including those associated with neuronneuron interactions (Neural cell adhesion molecule 1) and neuron-glial cell interactions (Neurofascin), neurogenesis (Lyssencephaly-1 homologue A, Alpha-Internexin, Stathmin, Dihydropyrimidinase-related protein), neurite outgrowth (Neural cell adhesion molecule 1, Neurofascin), neuronal precursor proliferation (Lyssencephaly-1 homologue A), synaptogenesis and synaptic plasticity (Neurofascin and 14-3-3 protein gamma), axonal guidance (Neurofascin), axonal regeneration (Macrophage migration inhibitory factor) and myelination (Neurofascin). Significantly, the induction of a serine-threonine kinase stathmin after SCI has already been demonstrated and it was associated with an increase in glial proliferation.^49^

In addition, several proteins with no known spinal function were identified (following a NCBI bibliographic database search), as well as Protein S100-B that has been proposed as a marker of SCI severity^46^ and Ubiquitin carboxyl-terminal hydrolase isozyme L1 that may be related to axon degradation.^50^ An upregulation of Gamma-synuclein has been described in the SC^51^ and the spinal dorsal horn^45^ although its precise role during acute SCI is not known. Moreover, both Thioredoxin-dependent peroxide reductase and Palmitoyl-protein thioesterase 1 have been linked to the negative regulation of neuron apoptosis (Swiss-prot Database). Finally, the Platelet-activating factor acetylhydrolase IB subunit alpha may promote the proliferation of neuronal precursors (Swiss-prot Database).

Taken together these data help highlight the change in the spinal cord proteome during acute and chronic SCI, as well helping to define the different profiles associated with symptoms such as neuropathic pain, spasticity, they will serve to benchmark future neuro-regenerative therapies. Despite the promising results obtained in these studies, it will be necessary to define more of the proteins present in the spinal cord proteome. We hope that by continuing these studies and complementing them, the characterization of the complete protein profile of the rat spinal cord will be possible, and differential expression analyses can be carried out in human and/or other animal models.

## Figures and Tables

**Table 1.** Spots identified with 2-DE gel (pH: 3-11 NL). The data indicates accession number, the isoelectric point (theoretical and experimental), molecular weight (theoretical and experimental), subcellular localization and recognised function.

**Protein name**	**Accession no.**	**MALDI-TOF/Spot Nº**	**LC-MS Q-TRAP**	**MW Da theorical**	**MW Da experimental**	**pI theorical**	**pI experimental**	**Subcellular localization**	**Function**
Myelin basic protein S	MBP_RAT	Spot Nº 2	Identified	21,489.00	15	11.25	10.8	Cell mb.	Structural…
Tubulin polymerization-promoting protein family	TPPP3_BOVIN	Spot Nº 3	Identified	18,931.00	24.5	9.18	10.8	Myelin mb.	Structural…
Protein NipSnap homolog 1	NIPS1_MOUSE	Spot Nº 6	No	33,342.00	35	9.48	10.8	Mit inn mb.	Others.
Prohibitin-2	PHB2_MOUSE	Spot Nº 8	No	33,276.00	40	9.83	10.8	Mit inn mb; Cp; N.	Others.
Aspartate aminotransferase, mitochondrial	AATM_RAT	Spot Nº 10	Identified	47,284.00	51	9.13	10.8	Mit; Cell mb.	Metabolism.
Elongation factor 1-alpha 1	EF1A1_CRIGR	Spot Nº 11	No	50,109.00	56	9.10	10.8	Cp.	Prot regulation.
Profilin-1	PROF1_RAT	Spot Nº 13	Identified	14,948.00	13	8.46	9.6	Cp.	Structural…
Peptidyl-prolyl cis-trans isomerase A	PPIA_RAT	Spot Nº 16	Identified	17,863.00	18	8.34	9.5	Cp.	Protregulation.
Destrin OS	DEST_RAT	Spot Nº 17	Identified	18.522,00	20	8.19	9.2		Structural…
Cofilin-1	COF1_RAT	Spot Nº 18	Identified	18,521.00	23	8.22	9.2	N; Cp.	Structural…
Peroxiredoxin-1	PRDX1_RAT	Spot Nº 19	Identified	22,095.00	31	8.27	9.5	Cp; Mel.	Stress Resp...
Peroxiredoxin-1	PRDX1_RAT	Spot Nº 20	Identified	22,095.00	31	8.27	9.2	Cp; Mel.	Stress Resp...
Proteasome subunit beta type-1	PSB1_MOUSE	Spot Nº 21	Identified	26,355.00	33	7.67	9.4	Cp; N.	Prot regulation.
Glutathione S-transferase alpha-3	GSTA3_RAT	Spot Nº 22	No	25,303.00	35	8.78	9.6	Cp.	Stress Resp...
Glutathione S-transferase Mu 1	GSTM1_RAT	Spot Nº 23	No	25,897.00	34.2	8.27	9.4	Cp.	Stress Resp...
Proteasome subunit alpha type-7	PSA7_MOUSE	Spot Nº 24	Identified	27,838.00	35.5	8.59	9.6	Cp; N.	Prot regulation.
ATP synthase subunit alpha liver isoform, mitochondrial	ATPA2_BOVIN	Spot Nº 25	No	38,852.00	37	9.57	9.6	Mit inn mb.	Metabolism.
ATP synthase subunit gamma, mitochondrial	ATPG_RAT	Spot Nº 26	Identified	30,172.00	38	8.87	9.5	Mit inn mb.	Metabolism.
Voltage-dependent anion-selective channel protein 1	VDAC1_RABIT	Spot Nº 27	Identified	30,722.00	39	8.62	9.5	Mit out mb; Cell mb.	Stress Resp...
Malate dehydrogenase, mitochondrial	MDHM_RAT	Spot Nº 28	Identified	35,661.00	45	8.93	9.9	Mit.	Metabolism.
Malate dehydrogenase, mitochondrial	MDHM_RAT	Spot Nº 29	Identified	35,661.00	45	8.93	9.65	Mit.	Metabolism.
L-lactate dehydrogenase A chain	LDHA_RAT	Spot Nº 30	Identified	36,427.00	45	8.45	9.5	Cp.	Metabolism.
Malate dehydrogenase, mitochondrial	MDHM_RAT	Spot Nº 31	Identified	35,661.00	49	8.93	9.5	Mit.	Metabolism.
Malate dehydrogenase, mitochondrial	MDHM_RAT	Spot Nº 32	Identified	35,661.00	45	8.93	9.4	Mit.	Metabolism.
Glyceraldehyde-3-phosphate dehydrogenase	G3P_RAT	Spot Nº 33	Identified	35,787.00	49	8.44	9.4	Cp.	Metabolism.
Fructose-bisphosphate aldolase A	ALDOA_RAT	Spot Nº 34	Identified	39,327.00	51	8.31	9.4	Mit.	Metabolism.
Cytochrome b-c1 complex subunit 2, mitochondrial	QCR2_RAT	Spot Nº 35	Identified	48,366.00	53	9.16	9.5	Mit inn mb.	Metabolism.
2’,3’-cyclic-nucleotide 3’-phosphodiesterase	CN37_RAT	Spot Nº 36	Identified	47,239.00	60	9.03	9.4	Cell mb; Mel.	Metabolism.
Septin-7	SEPT7_RAT	Spot Nº 37	No	50,518.00	66	8.73	9.4	Cp.	Structural…
Glyceraldehyde-3-phosphate dehydrogenase	G3P_RAT	Spot Nº 38	Identified	35,787.00	49	8.44	9.2	Cp.	Metabolism.
Glyceraldehyde-3-phosphate dehydrogenase	G3P_RAT	Spot Nº 39	Identified	35,787.00	50	8.44	9.2	Cp.	Metabolism.
Fumarate hydratase, mitochondrial	FUMH_RAT	Spot Nº 41	No	54,429.00	60	9.06	9.2	Mit; Cp.	Metabolism.
ATP synthase subunit alpha, mitochondrial	ATPA_RAT	Spot Nº 42	Identified	59,717.00	75	9.22	9.1	Mit inn mb.	Metabolism.
T-complex protein 1 subunit eta	TCPH_PONAB	Spot Nº 43	No	59,329.00	80	7.55	9.3	Cp.	Prot regulation.
Phosphoglycerate kinase 1	PGK1_RAT	Spot Nº 44	Identified	44,510.00	53	8.02	9.1	Cp.	Metabolism.
Fumarate hydratase, mitochondrial	FUMH_RAT	Spot Nº 45	No	54,429.00	60	9.06	8.9	Mit; Cp.	Metabolism.
Citrate synthase, mitochondrial	CISY_RAT	Spot Nº 46	Identified	51,833.00	53	8.53	8.88	Mit.	Metabolism.
Isocitrate dehydrogenase [NAD] subunit beta,	IDH3B_RAT	Spot Nº 47	Identified	42,327.00	51	8.89	8.87	Mit.	Metabolism.
Fructose-bisphosphate aldolase A	ALDOA_RAT	Spot Nº 48	Identified	39,327.00	51	8.31	8.8	Mit.	Metabolism.
Glyceraldehyde-3-phosphate dehydrogenase	G3P_RAT	Spot Nº 49	Identified	35,787.00	49	8.44	8.9	Cp.	Metabolism.
Thiosulfate sulfurtransferase	THTR_RAT	Spot Nº 50	Identified	33,386.00	45	7.71	9.2	Mit.	Carrier.
Hydroxyacyl-coenzyme A dehydrogenase,	HCDH_RAT	Spot Nº 51	No	34,426.00	40	8.83	9.2	Mit.	Metabolism.
Voltage-dependent anion-selective channel protein 1	VDAC1_RABIT	Spot Nº 52	Identified	30,722.00	39	8.62	9.2	Mit out mb; Cell mb.	Stress Resp...
Superoxide dismutase [Mn], mitochondrial	SODM_RAT	Spot Nº 56	Identified	24,659.00	30	8.96	8.88	Mit.	Stress Resp...
Peptidyl-prolyl cis-trans isomerase A	PPIA_RAT	Spot Nº 57	Identified	17,863.00	18.3	8.34	8.9	Cp.	Prot regulation.
Peptidyl-prolyl cis-trans isomerase A	PPIA_RAT	Spot Nº 58	Identified	17,863.00	18.3	8.34	8.7	Cp.	Prot regulation.
Cofilin-2 OS=Homo sapiens	COF2_HUMAN	Spot Nº 59	Identified	18,725.00	24	7.66	8.2	N; Cp.	Structural…
Superoxide dismutase [Mn], mitochondrial	SODM_RAT	Spot Nº 60	Identified	24,659.00	30	8.96	8.3	Mit.	Stress Resp...
Adenylate kinase isoenzyme 1	KAD1_RAT	Spot Nº 60	No	21,570.00	31.5	7.66	8.5	Cp.	Metabolism.
Glutathione S-transferase P	GSTP1_RAT	Spot Nº 61	Identified	23,424.00	31.3	6.89	8.6		Stress Resp...
Peroxiredoxin-1	PRDX1_RAT	Spot Nº 62	Identified	22,095.00	30	8.27	8.7	Cp; Mel.	Stress Resp...
Superoxide dismutase [Mn], mitochondrial	SODM_RAT	Spot Nº 63	Identified	24,659.00	34	8.96	8.7	Mit.	Stress Resp...
Cytochrome b-c1 complex subunit Rieske	UCRI_RAT	Spot Nº 64	Identified	29,427.00	35	9.04	8.7	Mit inn mb.	Metabolism.
Dihydropteridine reductase	DHPR_RAT	Spot Nº 65	No	25,536.00	36	7.67	8.7		Stress Resp...
Electron transfer flavoprotein subunit beta	ETFB_RAT	Spot Nº 66	Identified	27,670.00	39	7.60	8.8	Mit.	Metabolism.
Voltage-dependent anion-selective channel protein 1	VDAC1_RABIT	Spot Nº 67	Identified	30,722.00	49	8.62	8.8	Mit out mb; Cell mb.	Stress Resp...
Phosphoserine aminotransferase	SERC_HUMAN	Spot Nº 69	No	40,397.00	51	7.56	8.6	mb, mithoc	Metabolism.
Isocitrate dehydrogenase [NAD] subunit beta,	IDH3B_RAT	Spot Nº 70	Identified	42,327.00	53	8.89	8.6	Mit.	Metabolism.
Fructose-bisphosphate aldolase C	ALDOC_RAT	SpotNº 70	Identified	39,259.00	60	6.67	8.6		Metabolism.
Creatine kinase, ubiquitous mitochondrial	KCRU_MOUSE	Spot Nº 71	Identified	46,974.00	57	8.39	8.7	Mit inn mb.	Metabolism.
Phosphoglycerate kinase 1	PGK1_HORSE	Spot Nº 71	No	42,327.00	57	8.89	8.5		Metabolism.
Fumarate hydratase, mitochondrial	FUMH_RAT	Spot Nº 72	No	54,429.00	64	9.06	8.3	Mit; Cp.	Metabolism.
Pyruvate kinase isozymes M1/M2	KPYM_RAT	Spot Nº 73	Identified	57,781.00	90	6.63	8.2		Metabolism.
Transketolase	TKT_RAT	Spot Nº 74	Identified	67,601.00	84	7.23	8.2		Prot regulation.
Aconitate hydratase, mitochondrial	ACON_RAT	Spot Nº 75	Identified	85,380.00	80	7.87	8.2	Mit.	Metabolism.
Aconitate hydratase, mitochondrial	ACON_RAT	Spot Nº 76	Identified	85,380.00	78	7.87	8.3	Mit.	Metabolism.
Transketolase	TKT_RAT	Spot Nº 77	Identified	67,601.00	75	7.23	8.1		Prot regulation.
Pyruvate kinase isozymes M1/M2	KPYM_RAT	Spot Nº 78	Identified	57,781.00	75	6.63	8.0		Metabolism.
Glucose-6-phosphate isomerase	G6PI_RAT	Spot Nº 79	No	62,787.00	65	7.38	8.1	Cp.	Metabolism.
Glutamate dehydrogenase 1, mitochondrial	DHE3_RAT	Spot Nº 80	Identified	61,298.00	60	8.05	8.2	Mit.	Metabolism.
Glutamate dehydrogenase 1, mitochondrial	DHE3_RAT	Spot Nº 81	Identified	61,298.00	74	8.05	7.5	Mit.	Metabolism.
Vesicle-fusing ATPase	NSF_MOUSE	Spot Nº 82	Identified	82,561.00	52	6.52	8.2	Cp.	Prot regulation.
Platelet-activating factor acetylhydrolase IB subunit	LIS1_MOUSE	Spot Nº 83	Identified	46,670.00	53	6.97	8.0	Cp; N.	Structural…
Creatine kinase, ubiquitous mitochondrial	KCRU_MOUSE	Spot Nº 84	Identified	46,974.00	50	8.39	8.1	Mit inn mb.	Metabolism.
Aspartate aminotransferase, cytoplasmic	AATC_RAT	Spot Nº 85	Identified	46,400.00	49	6.73	8.2	Cp.	Metabolism.
Glutamine synthetase	GLNA_RAT	Spot Nº 86	Identified	42,240.00	49	6.64	8.05	Cp.	Metabolism.
Fructose-bisphosphate aldolase C	ALDOC_RAT	Spot Nº 87	Identified	39,259.00	50	6.67	8.0		Metabolism.
Glyceraldehyde-3-phosphate dehydrogenase	G3P_RAT	Spot Nº 88	Identified	35,787.00	51	8.44	7.9	Cp.	Metabolism.
Glyceraldehyde-3-phosphate dehydrogenase	G3P_RAT	Spot Nº 89	Identified	35,787.00	53	8.44	7.7	Cp.	Metabolism.
Alcohol dehydrogenase [NADP+]	AK1A1_RAT	Spot Nº 90	Identified	36,483.00	51	6.84	7.7		Stress Resp...
Isocitrate dehydrogenase [NADP] cytoplasmic	IDHC_RAT	Spot Nº 92	No	46,705.00	49	6.53	7.5	Cp.	Metabolism.
NAD-dependent deacetylase sirtuin-2	SIRT2_RAT	Spot Nº 95	Identified	39,294.00	36	6.67	8.1	Cp.	Structural…
Ribose-phosphate pyrophosphokinase 1	PRPS1_HUMAN	Spot Nº 96	No	34,812.00	36	6.51	7.8		Metabolism.
Electron transfer flavoprotein subunit alpha	ETFA_RAT	Spot Nº 97	No	34,929.00	35.5	8.62	7.7	Mit.	Metabolism.
Carbonic anhydrase 2	CAH2_RAT	Spot Nº 98	No	29,096.00	35	6.89	8.3	Cp.	Stress Resp...
Hydroxyacylglutathione hydrolase	GLO2_RAT	Spot Nº 99	Identified	28,878.00	34.1	6.46	8.2		Metabolism.
Phosphoglycerate mutase 1	PGAM1_MOUSE	Spot Nº 100	No	28,786.00	34	6.67	8.25	N.	Metabolism.
Triosephosphate isomerase	TPIS_RAT	Spot Nº 101	Identified	26,832.00	34	6.89	8.3		Metabolism.
Protein-L-isoaspartate (D-aspartate)	PIMT_RAT	Spot Nº 103	No	24,619.00	34	7.10	7.8	Cp.	Metabolism.
Glutathione S-transferase Yb-3	GSTM4_RAT	Spot Nº 104	No	25,664.00	35	6.84	7.75	Cp.	Stress Resp...
GTP-binding nuclear protein Ran	RAN_CANFA	Spot Nº 105	No	24,408.00	32	7.01	7.8	Cp; N; Mel.	Prot regulation.
Proteasome subunit alpha type-2	PSA2_RAT	Spot Nº 106	No	25,909.00	28	8.39	8.0	Cp; N.	Prot regulation.
Alpha-crystallin B chain	CRYAB_RAT	Spot Nº 109	Identified	20,076.00	18	6.76	8.2		Stress Resp...
Nucleoside diphosphate kinase B	NDKB_RAT	Spot Nº 110	No	17,272.00	8	6.92	8.3	Cp; Cell mb.	Metabolism.
Peroxiredoxin-5, mitochondrial	PRDX5_RAT	Spot Nº 111, 112	Identified	22,165.00	10	8.94	7.5	Mit; Cp; Per.	Stress Resp...
Peroxiredoxin-5, mitochondrial	PRDX5_RAT	Spot Nº 111, 112	Identified	22,165.00	11	8.94	7.0	Mit; Cp; Per.	Stress Resp...
Macrophage migration inhibitory factor	MIF_RAT	Spot Nº 113	Identified	12,496.00	15	6.79	7.1	Cp; ES; N.	Stress Resp...
Cytochrome c oxidase polypeptide 6A1, mitochondrial	CX6A1_CANFA	Spot Nº 114	No	2,109.00	15	6.48	7.3	Mit inn mb.	Metabolism.
D-dopachrome decarboxylase OS	DOPD_RAT	Spot Nº 115	Identified	13,125.00	15	6.09	6.8	Cp.	Stress Resp...
Histidine triad nucleotide-binding protein 1	HINT1_MOUSE	Spot Nº 116, 118	Identified	13,768.00	13.5	6.38	6.8	Cp.	Others.
Fatty acid-binding protein, epidermal OS	FABP5_RAT	Spot Nº 117	No	15,050.00	12.5	6.73	6.1	Cp.	Metabolism.
Histidine triad nucleotide-binding protein 1	HINT1_MOUSE	Spot Nº 116, 118	Identified	13,768.00	14	6.38	6.3	Cp.	Others.
Prefoldin subunit 1	PFD1_HUMAN	Spot Nº 119	Identified	14,202.00	20	6.32	6.3	???	Prot regulation.
Profilin-2	PROF2_RAT	Spot Nº 121	No	14,992.00	18.5	6.55	6.5	Cp.	Structural…
Nucleoside diphosphate kinase A	NDKA_RAT	Spot Nº 122	Identified	17,182.00	17	5.96	4.0	Cp; N.	Metabolism.
Superoxide dismutase [Cu-Zn]	SODC_RAT	Spot Nº 122	Identified	15,912.00	19	5.88	4.3	Cp.	Stress Resp...
Gamma-synuclein	SYUG_MOUSE	Spot Nº 125	Identified	13,152.00	19.5	4.63	5.2	Cp.	Structural…
Beta-synuclein	MNME_XYLFT	Spot Nº 126	Identified	14,268.00	22	4.43	4.7	Cp.	Structural…
Calmodulin	CALM_BOVIN	Spot Nº 127	Identified	16,827.00	31	4.09	3.6	Spindle.	Prot regulation.
Phosphatidylethanolamine-binding protein 1	PEBP1_RAT	Spot Nº 128	Identified	20,788.00	35	5.48	4.9	Cp; Cell mb.	Metabolism.
Peroxiredoxin-2	PRDX2_RAT	Spot Nº 129	Identified	21,765.00	35	5.20	4.7	Cp.	Stress Resp...
Peroxiredoxin-2	PRDX2_RAT	Spot Nº 129	Identified	21,765.00	32	5.20	4.3	Cp.	Stress Resp...
Ubiquitin carboxyl-terminal hydrolase isozyme L1	UCHL1_MOUSE	Spot Nº 131	Identified	24,822.00	32	5.14	4.5	Cp.	Prot regulation.
Rho GDP-dissociation inhibitor 1	GDIR1_RAT	Spot Nº 132	Identified	23,393.00	36	5.12	4.3	Cp.	Prot regulation.
Translationally-controlled tumor protein	TCTP_MOUSE	Spot Nº 133	Identified	19,450.00	38	4.76	4.2	Cp.	Structural…
Lactoylglutathione lyase	LGUL_RAT	Spot Nº 134	Identified	20,806.00	40	5.12	4.4		Stress Resp...
14-3-3 protein gamma	1433G_HUMAN	Spot Nº 135	Identified	28,235.00	40	4.80	4.5	Cp.	Prot regulation.
Calretinin	CALB2_RAT	Spot Nº 135	Identified	31,384.00	43	4.94	4.3		Carrier.
14-3-3 protein epsilon	1433E_BOVIN	Spot Nº 136	Identified	29,155.00	51	4.63	4.3	Cp; Mel.	Prot regulation.
Annexin A5	ANXA5_RAT	Spot Nº 137	Identified	35,722.00	53	4.93	4.4		Others.
Annexin A5	ANXA5_RAT	Spot Nº 138	Identified	35,722.00	75	4.93	3.88		Others.
Ubiquitin thioesterase OTUB1	OTUB1_RAT	Spot Nº 139	No	31,250.00	80	4.85	4.6		Prot regulation.
Glial fibrillary acidic protein	GFAP_RAT	Spot Nº 140	Identified	49,927.00	47	5.35	4.5	Cp.	Structural…
40S ribosomal protein SA	RSSA_RAT	Spot Nº 140	Identified	32,803.00	47	4.80	4.3	Cp.	Structural…
Glial fibrillary acidic protein	GFAP_RAT	Spot Nº 141	Identified	49,927.00	51	5.35	4.3	Cp.	Structural…
Calreticulin	CALR_RAT	Spot Nº 142	No	47,966.00	63	4.33	4.6	ER.	Prot regulation.
Rab GDP dissociation inhibitor alpha	GDIA_RAT	Spot Nº 143	Identified	50,504.00	64	5.00	5.0	Cp.	Carrier.
Heat shock protein HSP 90-beta	HS90B_RAT	Spot Nº 144	Identified	83,229.00	97	4.97	5.2	Cp; Mel.	Prot regulation.
Neurofilament medium polypeptide	NFM_RAT	Spot Nº 145	Identified	95,734.00	115	4.77	5.18		Structural…
Neurofilament medium polypeptide	NFM_RAT	Spot Nº 145	Identified	95,734.00	115	4.77	5.5		Structural…
Neurofilament heavy polypeptide	NFH_RAT	Spot Nº 146	Identified	115,308.00	160	5.74	5.2		Structural…
Gamma-enolase	ENOG_RAT	Spot Nº 147	Identified	47,111.00	66	5.03	5.3	Cp; Cell mb.	Metabolism.
Actin, cytoplasmic 1	ACT5_CHICK	Spot Nº 148	No	41,809.00	58	5.30	5.3	Cp.	Structural…
Tropomodulin-2	TMOD2_MOUSE	Spot Nº 149	Identified	39,487.00	57	5.28	5.6	Cp.	Structural…
Tubulin alpha-1 chain (Fragment)	TBA1_CHICK	Spot Nº 150	Identified	50,104.00	50	4.96	6.0		Structural…
L-lactate dehydrogenase B chain	LDHB_RAT	Spot Nº 151	Identified	36,589.00	51	5.70	5.7	Cp.	Metabolism.
Pyruvate dehydrogenase E1 component subunit beta, mitochondrial	ODPB_MOUSE	Spot Nº 152	Identified	38,912.00	49	6.41	6.0	Mit.	Metabolism.
Pyruvate dehydrogenase E1 component subunit beta, mitochondrial	ODPB_MOUSE	Spot Nº 154	Identified	38,912.00	45	6.41	6.4	Mit.	Metabolism.
L-lactate dehydrogenase B chain	LDHB_RAT	Spot Nº 155	Identified	36,589.00	45	5.70	6.8	Cp.	Metabolism.
L-lactate dehydrogenase B chain	LDHB_RAT	Spot Nº 158	Identified	36,589.00	36	5.70	5.5	Cp.	Metabolism.
Malate dehydrogenase, cytoplasmic	MDHC_RAT	Spot Nº 159	Identified	36,460.00	52	6.16	5.9	Cp.	Metabolism.
Malate dehydrogenase, cytoplasmic	MDHC_RAT	Spot Nº 160	Identified	36,460.00	33	6.16	5.7	Cp.	Metabolism.
Prohibitin	PHB_RAT	Spot Nº 162	Identified	29,802.00	34	5.57	6.2	Mit inn mb.	Others.
6-phosphogluconolactonase	6PGL_RAT	Spot Nº 164	No	27,217.00	34	5.54	6.7		Metabolism.
Peroxiredoxin-6	PRDX6_RAT	Spot Nº 165	Identified	24,803.00	32.5	5.64	6.5	Cp; Lys.	Stress Resp...
Peroxiredoxin-6	PRDX6_RAT	Spot Nº 165	Identified	24,803.00	32.5	5.64	6.7	Cp; Lys.	Stress Resp...
NADH dehydrogenase [ubiquinone] iron-sulfur protein 3, mitochondrial	NDUS3_MOUSE	Spot Nº 166	Identified	30,131.00	31.5	6.67	6.65	Mit inn mb.	Metabolism.
Protein DJ-1	PARK7_RAT	Spot Nº 167	Identified	19,961.00	25	6.32	6.65	N; Cp.	Stress Resp...
Dynactin subunit 3	DCTN3_BOVIN	Spot Nº 168	Identified	21,178.00	30	5.39	6.6	Cp.	Structural…
Glutathione S-transferase A6	GSTA6_RAT	Spot Nº 170	No	25,791.00	32	5.90	7.3	Cp.	Stress Resp...
Thioredoxin-dependent peroxide reductase, mitochondrial	PRDX3_RAT	Spot Nº 171	Identified	28,277.00	32	7.14	7.6	Mit.	Stress Resp...
Thioredoxin-dependent peroxide reductase, mitochondrial	PRDX3_RAT	Spot Nº 171	Identified	28,277.00	35.5	7.14	7.3	Mit.	Stress Resp...
Protein DJ-1	PARK7_RAT	Spot Nº 173	Identified	19,961.00	34	6.32	7.2	N; Cp.	Stress Resp...
ATP synthase subunit d, mitochondrial	ATP5H_RAT	Spot Nº 175	Identified	18,752.00	34	6.17	6.9	Mit inn mb.	Metabolism.
Protein-L-isoaspartate (D-aspartate) O-methyltransferase OS=Macaca fascicularis GN=PCMT1 PE=2 SV=3	PIMT_MACFA	Spot Nº 180	Identified	24,622.00	34	6.23	7.0	Cp.	Metabolism.
Flavin reductase	BLVRB_MOUSE	Spot Nº 181	No	22,183.00	35	6.49	7.15	Cp.	Stress Resp...
Protein-L-isoaspartate (D-aspartate) O-methyltransferase OS=Macaca fascicularis GN=PCMT1 PE=2 SV=3	PIMT_MACFA	Spot Nº 182	Identified	24,622.00	34	6.23	6.8	Cp.	Metabolism.
V-type proton ATPase subunit E 1	VATE1_BOVIN	Spot Nº 183	Identified	26,123.00	39	8.45	7.2		Metabolism.
Pirin	PIR_RAT	Spot Nº 184	No	32,158.00	40	6.22	7.5		Others.
NADH dehydrogenase [ubiquinone] 1 alpha subcomplex subunit 10, mitochondrial	NDUAA_RAT	Spot Nº 187	No	40,468.00	41	7.64	6.7	Mit.	Metabolism.
Elongation factor Tu, mitochondrial	EFTU_RAT	Spot Nº 190	Identified	49,491.00	75	7.23	7.1	Mit.	Prot regulation.
Alpha-enolase	ENOA_MOUSE	Spot Nº 191	Identified	47,111.00	80	6.37	7.8	Cp; Cell mb.	Metabolism.
Alpha-enolase	ENOA_MOUSE	Spot Nº 192	Identified	47,111.00	80	6.37	8.0	Cp; Cell mb.	Metabolism.
Beta-centractin	ACTY_MOUSE	Spot Nº 193	No	42,255.00	86	5.98	7.4	Cp.	Structural…
Alpha-enolase	ENOA_MOUSE	Spot Nº 194	Identified	47,111.00	80	6.37	6.5	Cp; Cell mb.	Metabolism.
Alpha-enolase	ENOA_MOUSE	Spot Nº 195	Identified	47,111.00	80	6.37	6.3	Cp; Cell mb.	Metabolism.
Syntaxin-binding protein 1	STXB1_BOVIN	Spot Nº 197	Identified	67,526.00	82	6.49	6.8	Cp; Cell mb.	Prot regulation.

**Abbreviations:** Cp, Cytoplasm; N, nucleus; Mit inn mb, mitochondrial inner membrane; Mit, mitochondrion; cell mb, cellular membrane; ES, extra cellular space; Mit out mb, mitochondrial outer membrane; Mel, Melanosome; Cp-sec-syn ves, Cytoplasmic-secreted-synaptic vesicle; Per mb, peroxisomal membrane; Lys, lysosome; Gol app, golgi apparatus; ER, endoplasmic reticulum; EM, extracellular matrix.

**Table 2.** Proteins identified with 1-D gel and LC-MS/MS analysis.

**Protein name**	**Accession no.**	**MW Da**	**pI**	**Subcellular localization**	**Function**
**Slice 2, 3**					
Protein S100-B	|P50114|S100B_MOUSEN	10728.05	4.52	Cp; N.	Carrier.
NADH dehydrogenase [ubiquinone] 1 alpha subcomplex subunit 4	|Q62425|NDUA4_MOUSE	9326.79	9.52	Mit inn mb.	Metabolism.
10 kDa heat shock protein, mitochondrial	s|P26772|CH10_RAT	10901.67	8.89	Mit.	Prot regulation.
Cytochrome c oxidase polypeptide 7A2, mitochondrial	sp|P35171|CX7A2_RAT	9352.97	10.28	Mit inn mb.	Metabolism.
Guanine nucleotide-binding protein G(I)/G(S)/G(O) subunit gamma-12	|Q9DAS9|GBG12_MOUSE	7997.23	9.14	Cell mb; Cp.	Carrier.
Cytochrome c oxidase subunit VIb isoform 1	|P56391|CX6B1_MOUSE	10071.45	8.96	Mit intermb sp.	Metabolism.
**Slice 4**					
ATP synthase subunit e, mitochondrial	sp|P29419|ATP5I_RAT	8254.65	9.34	Mit inn mb.	Metabolism.
Histone H2A type 1-A	|Q96QV6|H2A1A_HUMAN	14233.51	10.86	N.	Structural…
Acyl-CoA-binding protein	|P11030|ACBP_RAT	10027.46	8.78		Carrier.
Dynein light chain roadblock-type 1	|P62628|DLRB1_RATE	10989.68	6.58	Cp.	Structural…
Glutaredoxin-1	sp|Q9ESH6|GLRX1_RAT	11878.88	8.93	Cp.	Stress Resp...
Guanine nucleotide-binding protein G(I)/G(S)/G(O) subunit gamma-2	|P63213|GBG2_MOUSE	7850.14	7.78	Cell mb; Cp.	Carrier.
Glycogen phosphorylase, brain form	|P11216|PYGB_HUMAN	96695.96	6.40		Metabolism.
Mitochondrial import inner membrane translocase subunit Tim13	|Q9Y5L4|TIM13_HUMAN	10500.02	8.42	Mit inn mb.	Prot regulation.
Neurofilament light polypeptide	sp|P19527|NFL_RAT	61335.28	4.63		Structural…
**Slice 5**					
Galectin-1	|P16045|LEG1_MOUSE	14865.85	5.32	ES.	Others.
Cytochrome b-c1 complex subunit 7	|Q9D855|QCR7_MOUSE	13527.47	9.10	Mit inn mb.	Metabolism.
NADH dehydrogenase [ubiquinone] 1 alpha subcomplex subunit 5	sp|Q63362|NDUA5_RAT	13411.79	6.84	Mit inn mb.	Metabolism.
Ribonuclease UK114	|P52759|UK114_RAT	14303.46	7.80	Mit; Cp; N.	Prot regulation.
Myotrophin	|P62775|MTPN_RAT	12860.77	5.27	Cp.	Structural…
Thioredoxin	|P11232|THIO_RAT	11673.47	4.80	Cp.	Stress Resp...
Fatty acid-binding protein, brain	|P55051|FABP7_RAT	14863.98	5.46	Cp.	Carrier.
**Slice 6**					
Cytochrome c, somatic	|P62898|CYC_RAT	11605.44	9.61	Mit.	Metabolism.
Glial fibrillary acidic protein	lP47819 |GFAP_RAT	49957.09	5.35	Cp.	Structural…
CDGSH iron sulfur domain-containing protein 1	|Q9NZ45|CISD1_HUMAN	12199.05	9.20	Mit out mb.	Metabolism.
Parvalbumin alpha	|P02625|PRVA_RAT	11925.52	5.00		Carrier.
Astrocytic phosphoprotein PEA-15	|Q5U318|PEA15_RAT	15040.10	4.93	Cp.	Stress Resp...
**Slice 7**					
60S acidic ribosomal protein P2	sp|P02401|RLA2_RAT	11691.96	4.44		Prot regulation.
Myosin light polypeptide 6	sp|Q64119|MYL6_RAT	16975.15	4.46		Structural…
V-type proton ATPase subunit G 2	|Q9TSV6|VATG2_PIG	13579.34	10.26	Mel.	Carrier.
V-Vesicle-associated membrane protein 2	|P63045|VAMP2_RAT	12690.78	7.84	Cp-sec-syn ves.	Carrier.
Ubiquitin-conjugating enzyme E2 N	|Q9EQX9|UBE2N_RAT	17123.79	6.13		Prot regulation.
Histone H2A.J	|A9UMV8|H2AJ_RAT	14045.45	11.05	N.	Structural…
ATP synthase subunit delta, mitochondrial	|P35434|ATPD_RAT	17595.07	5.16	Mit inn mb.	Metabolism.
Calcineurin subunit B type 1	|P63100|CANB1_RAT	19299.91	4.64		Carrier.
Histone H2B type 2-E	|Q64524|H2B2E_MOUSE	13993.26	10.31	N.	Structural…
Vesicle-associated membrane protein 3	|Q4R8T0|VAMP3_MACFA	11319.13	8.89	Cell mb.	Prot regulation.
Single-stranded DNA-binding protein, mitochondrial	sp|P28042|SSB_RAT	17454.93	9.84	Mit.	Others.
Tubulin alpha-1A chain	|Q6AYZ1|TBA1C_RAT	50135.63	4.94		Structural…
Creatine kinase B-type	sp|Q04447|KCRB_MOUSE; sp|P07335|KCRB_RAT	42725.27	5.39	Cp.	Metabolism.
Fibrous sheath-interacting protein 1	|Q66H16|FSIP1_RAT	49568.06	5.02		Others.
Mitochondrial fission 1 protein	|Q9CQ92|FIS1_MOUSE	17008.65	8.55	Mit out mb; Per mb.	Stress Resp...
**Slice 8**					
Low molecular weight phosphotyrosine protein phosphatase	|Q5REM7|PPAC_PONAB	18086.50	6.29	Cp.	Prot regulation.
Peptidyl-prolyl cis-trans isomerase NIMA-interacting 1	|Q9QUR7|PIN1_MOUSE	18370.46	8.93	N.	Structural…
Protein S100-A16	|Q96FQ6|S10AG_HUMAN	11801.40	6.28		Carrier.
Vesicle-associated membrane protein 1	|Q63666|VAMP1_RAT	12796.81	6.24	Cp-sec-syn ves.	Prot regulation.
Histone H2B type 1-K	|Q8CGP1|H2B1K_MOUSE	13920.17	10.31	Nucleus.	Structural…
Visinin-like protein 1	|Q5RD22|VISL1_PONAB	22338.24	5.32		Prot regulation.
Thrombospondin type-1 domain-containing protein 7B	|Q6P4U0|THS7B_MOUSE	179309.14	8.01	Cell mb.	Others.
**Slice 9**					
ADP-ribosylation factor 3	|P61206|ARF3_RAT	20456.51	6.74	Gol app.	Prot regulation.
Prefoldin subunit 2	|B0BN18|PFD2_RAT	16579.73	6.20		Prot regulation.
Stathmin	|Q6DUB7|STMN1_PIG	17302.51	5.76	Cp.	Structural…
Vesicle-associated membrane protein-associated protein B	sp|A5GFS8|VAPB_PIG	27053.25	6.85	Cp ves.	Prot regulation.
Nucleoside diphosphate kinase A	|Q05982|NDKA_RAT	17192.74	5.96	Cp; N.	Metabolism.
Ubiquitin-conjugating enzyme E2 variant 2	|Q7M767|UB2V2_RAT	16352.71	7.79		Prot regulation.
Transgelin-3	|Q5R6R2|TAGL3_PONAB	22472.64	6.84		Others.
Ubiquitin-conjugating enzyme E2 L3	|P68037|UB2L3_MOUSE	17861.58	8.68		Prot regulation.
Endothelin-1	sp|P22388|EDN1_RAT	23134.93	9.77	Sec.	Stress Resp...
NADH dehydrogenase [ubiquinone] 1 alpha subcomplex subunit 13	sp|Q95KV7|NDUAD_BOVIN	16673.39	9.22	Mit inn mb; N.	Stress Resp...
**Slice 10**					
Tubulin beta chain	sp|P02554|TBB_PIG	49860.95	4.78		Structural…
Actin-related protein 2/3 complex subunit 5-like protein	|Q9BPX5|ARP5L_HUMAN	17010.32	6.31	Cp.	Structural…
Hippocalcin-like protein 1	|P37235|HPCL1_HUMAN	22338.24	5.32		Others.
Neurocalcin-delta	|Q5PQN0|NCALD_RAT	22245.23	5.23		Others.
Transcription factor BTF3	sp|Q64152|BTF3_MOUSE	22030.81	9.52	N.	Others.
Ferritin heavy chain	|P19132|FRIH_RAT	21126.66	5.86		Others.
Cell division control protein 42 homolog	|Q8CFN2|CDC42_RAT	21258.61	6.16	Cell mb.	Stress Resp...
Phospholipid hydroperoxide glutathione peroxidase, nuclear	|Q91XR8|GPX42_RAT	29184.69	10.83	N.	Stress Resp...
60S ribosomal protein L12	|P35979|RL12_MOUSE	17804.56	9.48		Others.
**Slice 11**					
Ferritin light chain 1	sp|P02793|FRIL1_RAT	20748.50	5.99		Others.
Cysteine and glycine-rich protein 1	sp|P47875|CSRP1_RAT	20613.48	8.90	N.	Others.
NADH dehydrogenase [ubiquinone] 1 beta subcomplex subunit 10	|Q9DCS9|NDUBA_MOUSE	21023.81	8.19	Mit inn mb.	Metabolism.
Tubulin beta-2C chain	|Q6P9T8|TBB2C_RAT	49800.98	4.79		Structural…
**Slice 12**					
ATP synthase subunit O, mitochondrial	sp|Q06647|ATPO_RAT	23397.55	10.03	Mit inn mb.	Metabolism.
Glutathione S-transferase P	sp|P47954|GSTP1_CRIMI	23469.00	7.64		Stress Resp...
Adenylate kinase isoenzyme 1	sp|P39069|KAD1_RAT	21583.76	7.66	Cp.	Metabolism.
Ras-related protein Rab-1B	|Q9H0U4|RAB1B_HUMAN	22163.10	5.55	Cell mb; Cp.	Prot regulation.
Glycolipid transfer protein	|B0BNM9|GLTP_RAT	23703.65	6.90	Cp.	Carrier.
Ras-related protein Rab-11B	|O35509|RB11B_RAT	24488.50	5.64	Cell mb.	Carrier.
Histone H2A.x	sp|P27661|H2AX_MOUSE	15142.60	10.74	N.	Structural…
Ras-related protein Rab-7a	|P51150|RAB7A_MOUSE	23489.75	6.39	Mel.	Prot regulation.
Cell cycle exit and neuronal differentiation protein 1	|Q5FVI4|CEND_RAT	15043.01	9.01	Cell mb.	Structural…
UMP-CMP kinase	|Q9DBP5|KCY_MOUSE	22165.33	5.68	N. Cp.	Metabolism.
Apolipoprotein D	|P23593|APOD_RAT	21634.86	4.93	Sec.	Carrier.
Microtubule-actin cross-linking factor 1, isoform 4	|Q96PK2|MACF4_HUMAN	670150.80	5.20	Cp.	Structural…
GrpE protein homolog 1, mitochondrial	|Q99LP6|GRPE1_MOUSE	24307.02	8.58	Mit.	Prot regulation.
Transgelin-2	|Q9WVA4|TAGL2_MOUSE	22393.42	8.41		Others.
Ras-related protein Rap-1b	|Q62636|RAP1B_RAT	20824.79	5.65	Cell mb; Cp.	Prot regulation.
Glutathione S-transferase P 1	|P19157|GSTP1_MOUSE	23609.18	7.69		Stress Resp...
**Slice 13**					
Heat shock protein beta-1	|P14602|HSPB1_MOUSE	23013.85	6.12		Stress Resp...
Myelin-oligodendrocyte glycoprotein	|Q63345|MOG_RAT	27881.56	8.61	Cell mb.	Structural…
Glutathione S-transferase Y1	|Q00285|GSTMU_CRILO	25818.98	8.74	Cp.	Stress Resp...
Tumor protein D52	|Q62393|TPD52_MOUSE	20059.41	4.87		Structural…
Osteoclast-stimulating factor 1	|Q62422|OSTF1_MOUSE	23782.74	5.46	Cp.	Others.
UPF0568 protein C14orf166 homolog	|Q9CQE8|CN166_MOUSE	28152.21	6.40	N; Cp.	Others.
NADH dehydrogenase [ubiquinone] flavoprotein 2, mitochondrial	|P19234|NDUV2_RAT	27378.34	6.23	Mit inn mb.	Metabolism.
3-hydroxyacyl-CoA dehydrogenase type-2	|O02691|HCD2_BOVIN	27140.29	8.45	Mit.	Others.
Glutathione S-transferase alpha I	|Q08863|GSTA1_RABIT	25691.11	8.92	Cp.	Stress Resp...
Ras-related protein Rab-5A	|P20339|RAB5A_HUMAN	23658.68	8.23	Cell mb; Mel.	Prot regulation.
**Slice 14**					
14-3-3 protein zeta/delta	|P63102|1433Z_RAT; sp|P63101|1433Z_MOUSE	27771.14	4.73	Cp; Mel.	Prot regulation.
Dihydropteridine reductase	|P11348|DHPR_RAT	25552.20	7.67		Stress Resp...
Succinate dehydrogenase [ubiquinone] iron-sulfur subunit, mitochondrial	|P21913|DHSB_RAT	31829.94	8.96	Mit inn mb.	Metabolism.
Gamma-enolase	|P09104|ENOG_HUMAN	47268.58	4.91	Cp; Cell mb.	Metabolism.
Tumor protein D54	|Q6PCT3|TPD54_RAT	23991.85	5.80		Prot regulation.
Coiled-coil-helix-coiled-coil-helix domain-containing protein 3, mitochondrial	|Q9CRB9|CHCH3_MOUSE	26334.52	8.56		Others.
14-3-3 protein eta	|P68511|1433F_RAT; sp|P68510|1433F_MOUSE; sp|P68509|1433F_BOVIN	28211.74	4.81	Cp.	Carrier.
Endoplasmic reticulum protein ERp29	|P52555|ERP29_RAT	28574.83	6.23	ER.	Prot regulation.
14-3-3 protein theta	|Q5ZMD1|1433T_CHICK	27782.28	4.68	Cp.	Prot regulation.
Proteasome subunit alpha type-5	|Q9Z2U1|PSA5_MOUSE	26411.03	4.74	Cp; N.	Prot regulation.
Electron transfer flavoprotein subunit beta	sp|Q68FU3|ETFB_RAT	27687.42	7.61	Mit.	Metabolism.
14-3-3 protein beta/alpha	|P35213|1433B_RAT	28054.39	4.81	Cp; Mel.	Prot regulation.
Myelin P0 protein	|Q6WEB5|MYP0_HORSE	27570.67	9.40	Cell mb.	Structural…
ADP/ATP translocase 1	|P48962|ADT1_MOUSE	32904.27	9.73	Mit inn mb.	Carrier.
Hypoxanthine-guanine phosphoribosyltransferase (Fragment)	|P00493|HPRT_MOUSE	24081.78	5.74	Cp.	Metabolism.
Acidic leucine-rich nuclear phosphoprotein 32 family member A	|P49911|AN32A_RAT	28564.59	3.99	N; Cp.	Stress Resp...
Cytochrome c1, heme protein, mitochondrial	sp|P00125|CY1_BOVIN	35296.75	9.14	Mit inn mb.	Metabolism.
Microtubule-associated protein 1A	|Q9QYR6|MAP1A_MOUSE	300139.96	4.92		Structural…
N(G),N(G)-dimethylarginine dimethylaminohydrolase 2	|Q6MG60|DDAH2_RAT	29687.91	5.66		Metabolism.
Myelin proteolipid protein	|P60203|MYPR_RAT	30077.17	8.71	Cell mb.	Structural…
Tropomyosin alpha-3 chain	|P06753|TPM3_HUMAN	32818.79	4.68	Cp.	Structural…
Peflin	|Q641Z8|PEF1_RAT	30012.40	5.67	Cp; Cell mb.	Others.
Rap guanine nucleotide exchange factor-like 1	|Q68EF8|RPGFL_MOUSE	73695.33	5.84		Carrier.
Calcyclin-binding protein	|Q6AYK6|CYBP_RAT	26541.19	7.64	N; Cp.	Prot regulation.
**Slice 15**					
Tropomyosin alpha-1 chain	|P42639|TPM1_PIG	32680.56	4.69	Cp.	Structural…
Pyruvate dehydrogenase E1 component subunit beta, mitochondrial	|P49432|ODPB_RAT	38982.13	6.20	Mit.	Metabolism.
3-hydroxyisobutyrate dehydrogenase, mitochondrial	|P29266|3HIDH_RAT	35302.71	8.73	Mit.	
Carbonyl reductase [NADPH] 1	|P47727|CBR1_RAT	30578.12	8.21	Cp.	Stress Resp...
EF-hand domain-containing protein D2	|A5D7A0|EFHD2_BOVIN	26918.43	5.26		Others.
Charged multivesicular body protein 4b	|Q9D8B3|CHM4B_MOUSE	24936.13	4.76	Cp.	Prot regulation.
Elongation factor 1-beta	|O70251|EF1B_MOUSE	24693.68	4.53		Prot regulation.
Clathrin light chain B	|P08082|CLCB_RAT	25117.44	4.56	Cp ves.	Prot regulation.
Complement component 1 Q subcomponent-binding protein, mitochondrial	|O35796|C1QBP_RAT	30996.92	4.77	Mit.	Others.
Methylglutaconyl-CoA hydratase, mitochondrial	|Q9JLZ3|AUHM_MOUSE	33394.99	9.56	Mit.	Metabolism.
Tropomyosin alpha-4 chain	|P09495|TPM4_RAT	28509.70	4.66	Cp.	Structural…
Coiled-coil-helix-coiled-coil-helix domain-containing protein 6	|Q91VN4|CHCH6_MOUSE	29798.81	8.41		Others.
Polymerase I and transcript release factor	|Q6NZI2|PTRF_HUMAN	43476.14	5.51	Cell mb; ER; Cp; Mit; N.	Others.
Drebrin-like protein	|Q9JHL4|DBNL_RAT	48612.51	4.89	Cp.	Structural…
Tubulin alpha-1B chain	|Q6P9V9|TBA1B_RAT	50151.63	4.94		Structural…
Syntaxin-1B	|P61265|STX1B_RAT	33244.69	5.25	Cell mb.	Carrier.
Clathrin light chain A	|P08081|CLCA_RAT	26980.50	4.41	Cp ves.	Prot regulation.
Phosphoglycerate kinase 2	|Q8MIF7|PGK2_HORSE	44879.16	8.62	Cp.	Metabolism.
Annexin A3	|P14669|ANXA3_RAT	36363.20	5.96		Others.
Acidic leucine-rich nuclear phosphoprotein 32 family member B	|Q9EST6|AN32B_RAT	31060.63	3.87	N.	Stress Resp...
Alpha-S1-casein	|O62823|CASA1_BUBBU	24326.77	4.87	Sec.	Carrier.
Tubulin beta chain	lP02554lTBB_PIG	49860.95	4.78		Structural…
**Slice 16**					
Apolipoprotein E	|P02650|APOE_RAT	35753.46	5.23	Sec.	Stress Resp...
Breast carcinoma-amplified sequence 1 homolog (Fragment)	|Q3ZB98|BCAS1_RAT	58623.87	5.58	Cp.	Others.
Heterogeneous nuclear ribonucleoproteins A2/B1	|O88569|ROA2_MOUSE	37402.67	8.97	N; Cp.	Others.
60S acidic ribosomal protein P0	sp|P19945|RLA0_RAT	34215.47	5.91		Prot regulation.
Adaptin ear-binding coat-associated protein 1	|P69682|NECP1_RAT	29792.40	5.97	Cp ves; Cell mb.	Prot regulation.
Alpha-internexin	|P23565|AINX_RAT	56115.38	5.20		Structural…
Gamma-soluble NSF attachment protein	|Q9CWZ7|SNAG_MOUSE	34732.33	5.31	Cell mb.	Prot regulation.
Heterogeneous nuclear ribonucleoprotein H3	|P31942|HNRH3_HUMAN	36926.49	6.37	N.	Others.
Elongation factor 1-delta	|P57776|EF1D_MOUSE	31293.03	4.91		Prot regulation.
RNA-binding protein Musashi homolog 2	|Q96DH6|MSI2H_HUMAN	39133.53	7.71	Cp; N.	Others.
Guanine nucleotide-binding protein G(I)/G(S)/G(T) subunit beta-1	|P54311|GBB1_RAT	37376.97	5.60		Carrier.
NSFL1 cofactor p47	p|O35987|NSF1C_RAT	40679.96	5.04	N; Gol app.	Others.
NADH dehydrogenase [ubiquinone] 1 alpha subcomplex subunit 9, mitochondrial	|Q9DC69|NDUA9_MOUSE	42509.15	9.75	Mit.	Metabolism.
F-actin-capping protein subunit alpha-1	|B2GUZ5|CAZA1_RAT	32909.77	5.34		Structural…
Putative heterogeneous nuclear ribonucleoprotein A1-like protein 3	|P0C7M2|RA1L3_HUMAN	34223.28	9.23	N; Cp.	Carrier.
Translocon-associated protein subunit alpha	|Q9CY50|SSRA_MOUSE	32065.01	4.36	ER:	Carrier.
Annexin A2	|Q07936|ANXA2_RAT	38678.24	7.55	Sec; EM; Mel.	Others.
Palmitoyl-protein thioesterase 1	|P45479|PPT1_RAT	34455.01	7.09	Lys.	Stress Resp...
Dihydropyrimidinase-related protein 2	|P47942|DPYL2_RAT	62277.57	5.95	Cp.	Others.
Putative L-aspartate dehydrogenase	|Q9DCQ2|ASPD_MOUSE	30269.63	6.45		Metabolism.
Transcriptional activator protein Pur-alpha	|P42669|PURA_MOUSE	34883.73	6.07	N.	Stress Resp...
**Slice 17**					
Tubulin beta-4 chain	|B4F7C2|B4F7C2_RAT	49585.77	4.78		Structural…
Tubulin alpha chain	|P68370|TBA1A_RAT	50630.14	4.93		Structural…
Vimentin	|P08670|VIME_HUMAN	53651.68	5.06		Structural…
Tubulin beta-3 chain	|Q4QRB4|TBB3_RAT	50418.65	4.82		Structural…
Citrate synthase, mitochondrial	|Q8VHF5|CISY_RAT	51866.75	8.54	Mit.	Metabolism.
Reticulocalbin-2	|Q8BP92|RCN2_MOUSE	37432.96	4.27	ER.	Others.
Septin-4	|Q4R4X5|SEPT4_MACFA	55147.26	5.64		Structural…
Protein kinase C and casein kinase substrate in neurons protein 1	|Q5R411|PACN1_PONAB	50921.58	5.15	Cp.	Structural…
Obg-like ATPase 1	|Q9NTK5|OLA1_HUMAN	44743.57	7.64		Metabolism.
Sodium/potassium-transporting ATPase subunit beta-1	|P07340|AT1B1_RAT	35201.59	8.83	Cell mb.	Carrier.
Hsc70-interacting protein	|P50503|F10A1_RAT	41279.50	5.28	Cp.	Prot regulation.
Dynactin subunit 2	|Q99KJ8|DCTN2_MOUSE	44116.88	5.14	Cp; Cell mb.	Structural…
Hsp90 co-chaperone Cdc37	|Q61081|CDC37_MOUSE	44510.36	5.24	Cp.	Prot regulation.
Creatine kinase, ubiquitous mitochondrial	|P30275|KCRU_MOUSE	47003.72	8.39	Mit inn mb.	Metabolism.
**Slice 18**					
Endophilin-A1	|O35179|SH3G2_RAT	39899.28	5.26	Cp; Cell mb.	Carrier.
F-box only protein 2	|Q80UW2|FBX2_MOUSE	33675.95	4.21		Prot regulation.
Septin-2	|Q91Y81|SEPT2_RAT	41592.55	6.15	Cp.	Structural…
Acetyl-CoA acetyltransferase, mitochondrial	|P17764|THIL_RAT	44695.00	8.92	Mit.	Metabolism.
Stomatin-like protein 2	|Q99JB2|STML2_MOUSE	38413.95	8.74	Cp; Cell mb.	Structural…
Fumarylacetoacetase	|A5PKH3|FAAA_BOVIN	45975.54	6.67		Metabolism.
NAD-dependent deacetylase sirtuin-2	|Q5RJQ4|SIRT2_RAT	39319.27	6.67	Cp.	Structural…
Acetyl-CoA acetyltransferase, cytosolic	|Q5XI22|THIC_RAT	41108.41	6.86	Cp.	Metabolism.
Thioredoxin-dependent peroxide reductase, mitochondrial	|P20108|PRDX3_MOUSE	28127.03	7.15	Mit.	Stress Resp...
Microtubule-associated protein 6	|Q63560|MAP6_RAT	100484.89	9.45	Cp; Gol app.	Structural…
Macrophage-capping protein	|Q6AYC4|CAPG_RAT	38798.86	6.11	N; Cp; Mel.	Structural…
Tubulin alpha-1D chain	|Q2HJ86|TBA1D_BOVIN	50282.78	4.91		Structural…
Heterogeneous nuclear ribonucleoprotein A3	|Q6URK4|ROA3_RAT	39651.99	9.10	N.	Others.
Neuromodulin	|P07936|NEUM_RAT	23603.34	4.61	Cell mb;	Structural…
Proto-oncogene C-crk	|Q63768|CRK_RAT	33844.72	5.39	Cp; Cell mb.	Others.
Sodium/potassium-transporting ATPase subunit beta-3	|Q63377|AT1B3_RAT	31829.68	8.08	Cell mb; Mel.	Others.
**Slice 19**					
60 kDa heat shock protein, mitochondrial	|P18687|CH60_CRIGR	60955.49	5.91	Mit.	Stress Resp...
Tubulin alpha-1C chain	|P68373|TBA1C_MOUSE	49937.37	4.96		Structural…
Peripherin	|P21807|PERI_RAT	53549.76	5.37		Structural…
Myc box-dependent-interacting protein 1	|O08839|BIN1_RAT	64533.21	4.95	Cp; N.	Structural…
Dihydrolipoyl dehydrogenase, mitochondrial	|Q6P6R2|DLDH_RAT	54038.09	7.96	Mit.	Metabolism.
Septin-8	|Q8CHH9|SEPT8_MOUSE	49812.39	5.68		Structural…
**Slice 20**					
Stress-70 protein, mitochondrial	|O35501|GRP75_CRIGR	73857.70	5.97	Mit.	Prot regulation.
Actin, cytoplasmic 2	|P63259|ACTG_RAT	41792.84	5.31	Cp.	Structural…
V-type proton ATPase catalytic subunit A	|P50516|VATA_MOUSE	68326.08	5.42		Metabolism.
Dihydrolipoyllysine-residue acetyltransferase component of pyruvate dehydrogenase complex, mitochondrial	|P08461|ODP2_RAT	67165.84	8.76	Mit.	Metabolism.
78 kDa glucose-regulated protein	|P06761|GRP78_RAT	72346.99	5.07	ER; Mel.	Carrier.
Annexin A6	|P48037|ANXA6_RAT	75754.16	5.39	Cp; Mel.	Others.
Lamin-A/C	|P48678|LMNA_MOUSE	74237.57	6.54	N.	Structural…
Myristoylated alanine-rich C-kinase substrate	|P29966|MARCS_HUMAN	29794.51	4.32	Cp; Cell mb.	Structural…
NADH-ubiquinone oxidoreductase 75 kDa subunit, mitochondrial	|Q66HF1|NDUS1_RAT	79412.33	5.65	Mit inn mb.	Metabolism.
Synaptotagmin-2	|P29101|SYT2_RAT	47209.57	8.18	Cp ves; syn ves.	Structural…
Heat shock-related 70 kDa protein 2	|P14659|HSP72_RAT	69641.66	5.51		Stress Resp...
Heat shock 70 kDa protein 12A	|Q8K0U4|HS12A_MOUSE	74978.38	6.32		Carrier.
**Slice 21**					
Heat shock protein HSP 90-alpha	sp|P46633|HS90A_CRIGR	84814.91	4.93		Stress Resp...
Mitochondrial inner membrane protein	sp|Q8CAQ8|IMMT_MOUSE	83900.08	6.18	Mit inn mb.	Others.
**Slice 22**					
Spectrin alpha chain, brain	|P16086|SPTA2_RAT	284637.50	5.20	Cp.	Structural…
Heat shock cognate 71 kDa protein	|P63018|HSP7C_RAT	70871.07	5.37	Cp. Mel.	Stress Resp...
Microtubule-associated protein 2	|P11137|MAP2_HUMAN	202410.75	4.77	Cp.	Structural…
Neural cell adhesion molecule 1	|P13595|NCAM1_MOUSE	94658.31	4.83	Cell mb.	Structural…
Rab GDP dissociation inhibitor alpha	|Q7YQM0|GDIA_PONPY	50536.64	5.00	Cp.	Carrier.
Dihydropyrimidinase-related protein 5	|Q9JHU0|DPYL5_RAT	61540.39	6.60	Cp.	Others.
Neurofascin	|Q810U3|NFASC_MOUSE	138004.21	5.79	Cell mb.	Structural…
**Slice 23**					
Tubulin beta-2A chain	|Q7TMM9|TBB2A_MOUSE	49906.97	4.78		Structural…
Tubulin alpha-1B chain	|Q6P9V9|TBA1B_RAT	50151.63	4.94		Structural…
Neuroblast differentiation-associated protein AHNAK	Q09666|AHNK_HUMAN	629101.22	5.80	N.	Others.
Regulating synaptic membrane exocytosis protein 1	|Q86UR5|RIMS1_HUMAN	179654.84	9.62	Cell mb.	Carrier.
**Slice 24**					
Sodium/potassium-transporting ATPase subunit alpha-3	|P06687|AT1A3_RAT	111691.53	5.26	Cell mb.	Carrier.
Aconitate hydratase, mitochondrial	|Q9ER34|ACON_RAT	85433.44	7.87	Mit.	Metabolism.
Tubulin beta-5 chain	|P09653|TBB5_CHICK	49670.82	4.78		Structural…
Myelin-associated glycoprotein	|P20917|MAG_MOUSE	69352.86	4.96	Cell mb.	Structural…
6-phosphofructokinase type C	|P47860|K6PP_RAT	85720.28	6.94		Metabolism.
Microtubule-associated protein 1B	|P15205|MAP1B_RAT	269499.65	4.74		Structural…
Plasma membrane calcium-transporting ATPase 2	|P11506|AT2B2_RAT	136811.20	5.70	Cell mb.	Metabolism.
Dihydrolipoyllysine-residue acetyltransferase component of pyruvate dehydrogenase complex, mitochondrial	|P08461|ODP2_RAT	67165.84	8.76	Mit.	Metabolism.
Microtubule-associated protein 1A	|P34926|MAP1A_RAT	299530.68	4.87		Structural…
Spectrin beta chain, brain 1	|Q62261|SPTB2_MOUSE	274223.06	5.40	Cp.	Structural…
Serine/threonine-protein phosphatase 2A 65 kDa regulatory subunit A alpha isoform	|Q76MZ3|2AAA_MOUSE	65322.61	5.00		Prot regulation.
Hexokinase-1	|P27595|HXK1_BOVIN	102408.01	6.29	Mit out mb.	Metabolism.
